# Circulating cell‐free epstein–barr virus DNA levels and clinical features in Moroccan patients with nasopharyngeal carcinoma

**DOI:** 10.1186/s13027-021-00353-8

**Published:** 2021-02-18

**Authors:** Amina Gihbid, Raja Benzeid, Abdellah Faouzi, Jalal Nourlil, Nezha Tawfiq, Nadia Benchakroun, Amal Guensi, Karima Bendahhou, Abdellatif Benider, Naima El Benna, Imane Chaoui, Rachida Cadi, Mohammed El Mzibri, Meriem Khyatti

**Affiliations:** 1 Laboratory of Viral Oncology, Institut Pasteur du Maroc, Casablanca, Morocco; 2 Laboratory of Pathophysiology, Molecular Genetics and Biotechnology, Faculty of Sciences Ain Chock, Hassan II University, Casablanca, Morocco; 3 Biology and Medical Research Unit, National Center of Energy, Sciences and Nuclear Techniques, Rabat, Morocco; 4 Laboratory of Medical Virology & BSL-3, Institut Pasteur du Maroc, Casablanca, Morocco; 5 Mohammed VI Center for Cancer Treatment, Ibn Rochd University Hospital, Casablanca, Morocco; 6 Nuclear Medicine Department, Ibn Rochd University Hospital, Hassan II University, Casablanca, Morocco; 7 Department of Radiology, Ibn Rochd University Hospital, Hopital 20 Août, Casablanca, Morocco

**Keywords:** Epstein‐barr virus, Plasma DNA load, Nasopharyngeal carcinoma, Prognostic

## Abstract

**Background:**

The identification of effective prognosis biomarkers for nasopharyngeal carcinoma (NPC) is crucial to improve treatment and patient outcomes. In the present study, we have attempted to evaluate the correlation between pre-treatment plasmatic Epstein-Barr virus (EBV) DNA load and the conventional prognostic factors in Moroccan patients with NPC.

**Methods:**

The present study was conducted on 121 histologically confirmed NPC patients, recruited from January 2017 to December 2018. Circulating levels of EBV DNA were measured before therapy initiation using real-time quantitative PCR.

**Results:**

Overall, undifferentiated non-keratinizingcarcinoma type was the most common histological type (90.1 %), and 61.8 % of patients were diagnosed at an advanced disease stage (IV). Results of pre-treatment plasma EBV load showed that 90.9 % of patients had detectable EBV DNA, with a median plasmatic viral load of 7710 IU/ml. The correlation between pre-treatment EBV DNA load and the conventional prognostic factors showed a significant association with patients’ age (*p* = 0.01), tumor classification (*p* = 0.01), lymph node status (*p* = 0.003), metastasis status (*p* = 0.00) and overall cancer stage (*p* = 0.01). Unexpectedly, a significant higher level of pre-treatment EBV DNA was also found in plasma of NPC patients with a family history of cancer (*p* = 0.04). The risk of NPC mortality in patients with high pretreatment EBVDNA levels was significantly higher than that of those with low pre-treatment plasma EBV-DNA levels (*p* < 0.05). Furthermore, patients with high pre-treatment EBV-DNA levels (≥ 2000, ≥ 4000) had a significant low overall survival (OS) rates (*p* < 0.05). Interestingly, lymph node involvement, metastasis status and OS were found to be the most important factors influencing the EBV DNA load in NPC patients.

**Conclusions:**

The results of the present study clearly showed a high association between pre-treatment EBV DNA load, the crucial classical prognostic factors (T, N, M and disease stage) of NPC and OS, suggesting that pre-treatment EBV DNA can be a useful prognostic biomarker in clinical decision-making and improving NPC treatment in Morocco.

## Introduction

Nasopharyngeal carcinoma (NPC), one of the most common head and neck cancers, has a streaking geographical distribution; rare in most parts of the world, it is endemic in southern parts of China and other parts of south-east Asia (Malaysia, Indonesia, Vietnam), with an incidence varying between 30 and 80 cases per 100,000 per year. Intermediate rates are also observed in certain areas including the Maghreb (Morocco, Algeria & Tunisia), the Inuit of Greenland and Alaska [1, 2]. In Morocco, according to the cancer registry of Casablanca, the incidence of NPC is 4.2/100.000 in men and 1.2 /100.000 in women yearly [3]. The age incidence curves for NPC are different across endemic and non-endemic populations. A bimodal age distribution characterizes the population of the Maghreb, with a first peak incidence occurring at 15–25 and a second one at 50–59 years of age, while only one peak around ages 45–59 is observed in endemic areas for NPC [4].

NPC development seems to be multifactorial, in which ethnic background, genetic susceptibility and environmental carcinogens play a crucial role [5]. The strong association between Epstein Barr virus (EBV) and NPC implies that targeting EBV may be an efficient approach at different levels of NPC management. As such, EBV infection remains the most extensively studied etiological factor for NPC.

Several prognostic factors including clinical, biological and pathological data are widely used to characterize NPC evolution. In clinical practice, oncologists often rely on age, histological type and clinical stage as essential factors to predict the prognosis of NPC [6].

Currently, new prognostic biomarkers are being evaluated for their clinical involvement. Interestingly, the quantification of circulating EBV DNA represents one of the most sensitive peripheral blood biomarkers for NPC management [7]. Results from earlier studies demonstrate a strong association between tumor burden and pre-treatment EBV DNA load [8], the latter can also be useful to predict relapse and distant metastasis, which are the main challenge faced by clinicians in NPC management [9]. Moreover, many studies suggested that plasma EBV DNA level could improve and supplement TNM staging of NPC [10]. Accordingly, the clinical utility of this biomarker in screening, diagnostic and prognostication of NPC was largely highlighted in endemic areas for NPC (Asian populations) [11, 12].

Taking into account the biological variation between populations, which may influence the EBV DNA load, and the limited number of studies examining the clinical value of pre-treatment EBV DNA load in intermediate and non-endemic areas of NPC, this prospective study was designed to evaluate the correlation between pre-treatment plasmatic EBV DNA load, the conventional prognostic factors and patient’s survival in order to assess their clinical value in Moroccan NPC patients for better management of NPC.

## Materials & methods

### Study setting

A total of 121 histologically confirmed NPC patients were recruited at Mohammed IV Center for Treatment of Cancer of Casablanca, from January 2017 to December 2018. Sixty healthy individuals with no evidence of any personal history of cancer or other chronic disease were used as controls. The recruited healthy controls were matched to NPC cases by age and sex. The study protocol was approved by the Ethics Committee of Ibn Rochd Hospital of Casablanca, Morocco and written informed consent was obtained from each patient.

### Study design

At recruitment, all patients underwent a pre-treatment examination. Magnetic resonance imaging (MRI) of the neck and nasopharynx and Positron Emission Tomography–Computed Tomography (PET-CT) were performed for patients before the initiation of oncological therapy. All patients were staged according to the 7th edition of the International Union Against Cancer / American Joint Committee on Cancer system using MRI and PET/CT findings. A face-to- face interview was conducted with all participants to collect socio-economic information regarding: age, sex, familial history of cancer, tobacco habits, alcohol consumption and childhood habitat. Clinical information including histological type and clinical stage were retrieved from patients’ medical records. Patients included in this study were treated with chemo-radiotherapy with or without induction chemotherapy, according to the hospital practice guidelines. Patients as well as healthy individuals were subjected to peripheral blood sampling before initiation of treatment for plasmatic cell-free DNA extraction and EBV DNA quantification.

### Quantification of plasmatic EBV DNA

Plasma from NPC patients was recovered and conserved at -80 °C until use. A volume of 200 µl of plasma was used to extract DNA using QIAamp DNA Mini Kit (Qiagen, France). Circulating levels of EBV DNA were measured by real-time quantitative polymerase chain reaction (q-PCR) and were expressed as International Unit/ml (IU/ml). The q-PCR reaction was performed using the RealStar® EBV PCR Kit 1.0 (Altona Diagnostics GmbH, Germany) on ABI Prism 7700 sequence detection system (Applied Biosystems). The thermal cycling conditions were 95 °C for 10 min and 45 cycles of 95 °C for 15 sec, 58 °C for 1 min, 68 °C for 30 sec. For each run, two negative controls without any DNA template were included.

### Statistical analysis

Statistical analyses were performed using the Statistical Package for the Social Sciences (SPSS) version 22.0 for Windows. Descriptive statistics were calculated and presented as number and percentages. The Kruskal-Wallis H and the Mann–Whitney U tests were applied to study the association between classical prognostic factors and pre-treatment EBV viral load as appropriate. Overall survival (OS) was defined as the time from random assignment until death or loss to follow-up. The mortality rates of each group of patients were tested by the χ2 test. Three-year survival rates were estimated, and the periods of OS among the different groups of patients were evaluated by the Kaplan-Meier method and analyzed by the log-rank test. Differences were considered significant when *p* values were less than 0.05.

## Results

The socio-economic and clinico-pathologic data of the 121 NPC patients enrolled in this study are reported in Table 1 and showed that 76 were men and 45 were women (sex-ratio: 1.7). The median age of patients was 45 years with extreme ages of 12 and 80 years old. The proportion of young patients ≤ 30 years old was 24.8 %. The distribution of patients according to birth area showed that 43.3 % were from rural areas whereas 56.2 % were from urban areas. Of note, 22.3 % of patients had a family history of cancer.

**Table 1:** Socio-economic and clinico-pathologic characteristics of patients recruited in the study.

Characteristics	Number of cases	%
**Age**		
≤ 30 years	30/121	24.8
> 30 years	91/121	75.2
**Gender**		
Female	45/121	37.2
Male	76/121	62.8
**Gender and Age**		
Female, ≤ 30	16/121	13.2
Female, > 30	29/121	23.9
Male, ≤ 30	17/121	14.0
Male, > 30	59/121	48.7
**Childhood habitat**		
Rural	53/121	43.3
Urban	68/121	56.2
**Family history of cancer**		
No	94/121	77.7
Yes	27/121	22.3
**Histological type**		
Type 1	1/121	0.8
Type 2	8/121	6.6
Type 3	109/121	90.1
Others	3/121	2.5
**TNM classification**		
T1-T2	33/118	27.9
T3-T4	85/118	72.0
N0-N1	47/118	39.8
N2-N3	71/118	60.2
**Metastasis status**		
M0	80/118	67.7
M1	38/118	32.2
**Stage of the disease**		
I	3/118	2.5
II	15/118	12.7
III	27/118	22.8
IV	73/118	61.8
IVA	30/118	25.4
IVB	5/118	4.2
IVC	38/118	32.2

The World Health Organization (WHO) has classified NPC into the three subtypes based on histology: the keratinizing squamous cell carcinoma (type 1), the differentiated non-keratinizing carcinoma (type 2), and the undifferentiated non-keratinizing carcinoma (type 3). Pathological data showed that the most frequent histopathologic type was the undifferentiated non-keratinizing carcinoma (type 3) found in 90.1 % of cases. According to the available results of PET/CT and MRI, primary tumors of 27.9 % of patients were T1-T2, while those of 72.0 % of patients were T3-T4. Moreover, 39.8 % of patients had N0-N1 lymph-node involvement and 60.2 % N2-N3. Distant metastasis at initial diagnosis was detected in 32.2 % of patients. Clinical staging was as follows: 73 patients were classified at stage IV (61.8 %), 27 at stage III (22.8 %), 15 at stage II (12.7 %) and 3 at stage I (2.5 %).

Data of pre-treatment plasma EBV quantification showed that EBV DNA was detected in 110 out of 121 patients (90.9 % of cases) with an overall median plasmatic viral load of 7710 IU/ml [0– 2,742,000]. Furthermore, EBV DNA was detected in only one of the 60 healthy tested individuals, with an overall median plasmatic EBV load in the healthy group of 0 UI/ml [0–445 UI/ml]. The correlation between EBV DNA load and the conventional prognostic factors are reported in Table 2. In addition to the age and sex, largely considered as classical prognostic factors, correlations between cigarette smoking, alcohol consumption and family history of cancer and EBV DNA levels were also performed. Our results revealed a significant association between EBV DNA load and patient’s age (*p* = 0.01). In fact, the mean pre-treatment EBV DNA load was significantly low in young patients (24,694 IU/ml), while, it was considerably high in the plasma of patients older than 30 years (119,801 IU/ml). Regarding gender, although a minor variation was observed while comparing EBV DNA levels between men and women, the difference was statistically not significant (*p* = 0.5). Our data further showed that young females and young males had a low EBV DNA load as compared to older groups although the difference was not statically significant (*p* = 0.4). Moreover, statistical analysis revealed no significant difference between plasma EBV DNA load, cigarette smoking and alcohol consumption (*p* > 0.05). Unexpectedly, EBV DNA load was found to increase significantly in plasma of NPC patients with a family history of cancer (*p* = 0.04).

**Table 2:** Distribution of plasma EBV DNA loads according to conventional prognostic factors.

Characteristics	EBV DNA load		*p*-value
	**Mean (IU/ml)**	**σ**	
**Age**			
≤ 30	24,694	± 63,760	0.01
> 30	119,801	± 369,113	
**Gender**			
Female	62,539	± 186,538	0.1
Male	80,909	± 231,513	
**Gender and Age**			
Female, ≤ 30	42,658	± 93,241	
Female, > 30	70,876	± 214,835	0.4
Male, ≤ 30	10,957	± 19,628	
Male, > 30	101,064	± 259,527	
**Cigarette smoking**			
No	109,881	± 368,262	0.2
Yes	66,385	± 195,496	
**Alcohol consumption**			
No	95,116	± 324,783	0.2
Yes	106,263	± 328,960	
**Family history of cancer**			
No	63,627	± 160,216	0.04
Yes	209,697	± 612,347	
**Tumor classification**			
T1-T2	30,072	± 58,802	0.012
T3-T4	124,706	± 381,729	
**Lymph node status**			
N0-N1	32,175	± 85,392	0.003
N2-N3	141,974	± 412,092	
**Metastasis status**			
M0	30,455	± 67,107	0.00
M1	240,946	± 546,896	
**Stage of the disease**			
I	2197	± 3442	0.01
II	29,266	± 58,612	
III	29,515	± 77,795	
IV	141,779	± 476,377	

Interestingly, statistical analysis clearly showed that plasma EBV DNA loads correlate significantly with tumor classification (*p* = 0.007), lymph node status (*p* = 0.004), metastasis status (*p* = 0.00), and overall cancer stage (*p* = 0.01). EBV DNA levels were much higher in T3-T4 groups (124,706 IU/ml), N2-N3 groups (141,974 IU/ml) and in patients with distant metastasis (240,946 IU/ml), as compared to patients diagnosed in T1-T2 category (30,072 IU/ml), N0-N1 groups (32,175 IU/ml) and patients without metastasis (30,455 IU/ml), respectively.

Similarly, our data showed that plasma EBV DNA load correlates significantly with disease stage (*p* < 0.01). Indeed, corresponding plasma EBV DNA levels for clinical stages of NPC I, II, III and IV were 2197 IU/ml, 29,266 IU/ml, 29,515 IU/ml and 141,779 IU/ml, respectively. It is also noteworthy that EBV DNA loads of patients at stage IV were 60 times higher than those of patients at stage I.

**Fig. 1:**
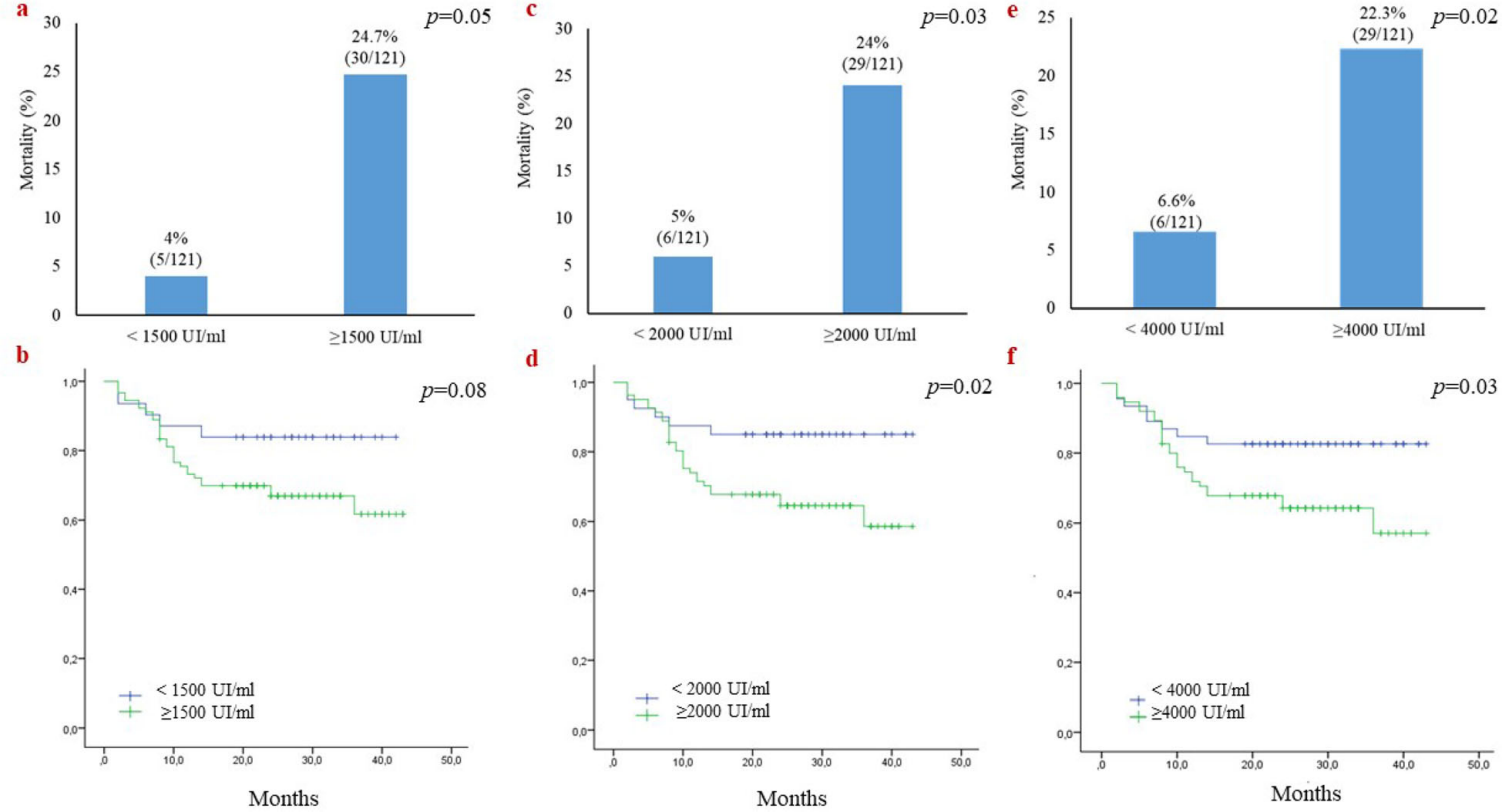
Mortality and overall survival of NPC patients according to their pre-treatment EBV DNA load levels. **a**, **b** pre-treatment EBV DNA load < 1500 vs.≥1500 IU/ml. **c**, **d** pre-treatment EBV DNA load < 2000 vs.≥2000 IU/ml. **e**, **f** pre-treatment EBV DNA load < 4000 vs.≥4000 IU/ml. The *p*-value corresponds to the log-rank test value

In the present study, survival rates were estimated for a 3-year period (2 years’ median period follow-up). Our results showed that pre-treatment EBV DNA load impacted significantly the OS; in fact, the risk of mortality in patients with high pretreatment EBVDNA levels was significantly higher compared to those with low pre-treatment plasma EBV-DNA levels (*p* < 0.05; Fig. 1a, c and e). The OS rate was 84 % among patients having an EBV DNA load less than 1500 IU/ml and 61 % among those with an EBV DNA load higher than 1500 copies/ml, although the difference was not statistically significant (*p* > 0.05; Fig. 1b). Further, patients with high pre-treatment EBV-DNA levels (≥ 2000 IU/ml or ≥ 4000 IU/ml) had a significant low overall survival (OS) rates (*p* < 0.05; Fig. 1d and f).

## Discussion

Over the past decades, several studies have supported that EBV DNA viral load represents one of the most sensitive and non-invasive biomarkers for NPC monitoring. In the present study, pre-treatment plasmatic EBV DNA loads of Moroccan NPC patients were quantified and assessed according to the conventional patient and clinical related prognostic factors and patient’s survival to evaluate their prognostic value. Overall, EBV DNA positivity was 90.9 %, which is comparable to that found in endemic areas [13], but higher than what was reported in non-endemic areas (79.2 %) [14]. Of particular interest, our results revealed a median plasmatic EBV viral load before treatment of 7710 IU/ml, which is comparable to previously findings among Tunisian and Chinese patients with NPC [15, 16]. Most of these previous studies revealed the potential value of this biomarker, whatever the geographic region or the ethnic group. In addition, comparative studies showed that plasmatic EBV DNA before treatment was superior to EBV IgA / VCA serum antibodies in the prognosis of NPC; in fact, studies reported that the levels of some antibodies remain high even when the patients were in complete remission [8, 17, 18].

Like all cancers, the most classical determining factors used to predict prognosis are age, sex, histological type and disease stage. As NPC is characterized by a bimodal age distribution in North-African countries, we analyzed the prognostic value of EBV DNA load in NPC patients stratified by age (≤ 30 vs. > 30 year of age). Our results showed a statistically significant difference between the two patients groups (*p* = 0.01); EBV DNA load was 4 times higher in patients older than 30 years old (119,801 IU/ml), compared to those less or equal to 30 years old (24,694 IU/ml). Our finding is consistent with previous studies showing that EBV replication is more active in older age groups with NPC [19–21].

TNM staging system has traditionally been the most important prognostic factor for NPC and it was largely established that EBV-DNA loads in NPC patients increased with disease stage. In this study, statistical analysis showed that pre-treatment EBV DNA levels correlate significantly with tumor, lymph node and metastatic status of our patients. The mean plasma EBV DNA levels were 124,706 IU/ml, 141,974 UI/ml and 240,946 UI/ml in patients with T3-T4, N3-N4 and distant metastasis, respectively, which is much higher than those obtained with T1-T2 (30,072 UI/ml), N1- N2 (32,175 IU/ml) and non-metastatic cases (30,455 UI/ml), respectively. These findings are in agreement with previous reports supporting the strong correlation between TNM staging and EBV DNA load and considering EBV DNA load as a useful tool to supplement the TNM system prognosis in NPC [14, 22].

In addition to the previous classical prognostic factors for NPC, we also evaluated the association between EBV DNA level and other factors incremented in other head and neck cancers. Cigarette smoking and alcohol drinking were reported to be associated with poor outcomes in patients with head-and-neck squamous cell carcinomas [23]. In our study, 31.4 % of patients were smokers and 9.9 % were alcohol consumers, but no significant differences of EBV DNA levels neither between smokers and non-smokers, nor between alcohol drinkers and non-drinkers were observed. Lv et al. have reported, in a large study involving 1051 NPC patients, that cigarette smoking and baseline plasma EBV DNA were independent prognostic factors for survival outcomes. In addition, the combined prognostic value of cigarette smoking and baseline plasma EBV DNA was found to be more significant than the individual factors [24].

Overall, 22.3 % of patients had a family history of cancer. It is also noteworthy that EBV DNA load was found to increase significantly in plasma of NPC patients with a family history of cancer (*p* = 0.04). This is in line with our recently published study using clustering algorithms and unexpectedly showing that familial history of cancer was strongly associated with advanced stage of NPC [25]. To the best of our knowledge, only one study, conducted on 1773 Chinese NPC patients, has examined the relationship between family history of cancer and prognosis of NPC, and has reported that EBV DNA levels were significantly higher in patients with family history of cancer, suggesting that these patients may have a genetic predisposition, promoting the biological process related to the initiation and progression of this disease, notably the activation of EBV viral cycle [26].

Regarding mortality and OS of NPC patients according to their pre-treatment EBV DNA load levels, our results showed patients with high pre-treatment EBV-DNA levels (≥ 2000, ≥ 4000) have significant high rates of mortality and low OS rates. A meta-analysis study on 7698 patients has confirmed that higher levels of EBV DNA before treatment were associated with higher risk of death (pooled hazard ratios (HR) = 2.420 (95 % confidence interval [CI] = 1.806–3.244, *p* < 0.001)), higher risk of disease progression (HR = 3.005 (95 % CI = 2.245–4.022; P < 0.001)) and higher risk of the occurrence of distant metastasis (HR = 3.684, 95 % CI = 3.055–4.444, *p* < 0.001) [13]. It is widely accepted that EBV DNA in the peripheral circulation of NPC patients is released from apoptotic and necrotic tumor cells and reflect the tumor burden [27]. Accordingly, EBV DNA becomes undetectable in NPC patients in complete remission and high pre- treatment plasma EBV DNA load correlates with poor prognosis [23].

The present study is very informative and clearly shows that EBV DNA load is higher in older patients and in patients with family history of cancer. Moreover, the level of EBV DNA is much higher according to tumor and clinical stages, lymph node status and metastatic status, whereas OS was impacted by a high levels of pre-treatment EBV DNA, suggesting that this EBV DNA load could be an effective and good prognostic biomarker of NPC in intermediate incidence risk area. However, the main limitations of this study is the small number of patients.

In Morocco, detection of EBV and assessment of EBV DNA load are not used as standard tests in the clinical practice of NPC. The present study highlights the value of plasma EBV DNA load quantification as useful biomarker, in combination with TNM staging, for NPC diagnosis and prognosis in Moroccan context.

## Conclusions

Globally, this study provides the first global report of EBV plasmatic viral load in NPC Moroccan patients. The outcomes highlight a very strong association between level of circulating EBV DNA, the classical prognostic factors and patient’s overall survival, suggesting that this biomarker could be predictive of the prognosis of NPC patients in intermediate endemic areas of NPC such as Morocco.

## Data Availability

The prospective data that support the findings of this study are available from the corresponding author upon reasonable request.
